# Contribution of Ghrelin to the Pathogenesis of Growth Hormone Deficiency

**DOI:** 10.3390/ijms22169066

**Published:** 2021-08-23

**Authors:** Andrzej Lewiński, Małgorzata Karbownik-Lewińska, Katarzyna Wieczorek-Szukała, Magdalena Stasiak, Renata Stawerska

**Affiliations:** 1 Department of Endocrinology and Metabolic Diseases, Medical University of Lodz, 93-338 Lodz, Poland; katarzyna.wieczorek@umed.lodz.pl; 2 Department of Endocrinology and Metabolic Diseases, Polish Mother’s Memorial Hospital—Research Institute, 93-338 Lodz, Poland; mkarbownik@hotmail.com (M.K.-L.); mstasiak33@gmail.com (M.S.); renata.stawerska@umed.lodz.pl (R.S.); 3 Department of Oncological Endocrinology, Medical University of Lodz, 90-419 Lodz, Poland; 4 Department of Paediatric Endocrinology, Medical University of Lodz, 90-419 Lodz, Poland

**Keywords:** growth hormone, growth hormone deficiency, ghrelin, IGF-1, *Helicobacter pylori*, intestinal microflora, thyroid hormones

## Abstract

In this review we described the interactions between ghrelin and the growth hormone (GH)-insulin-like growth factor 1 (IGF-1) axis in children and adults with growth hormone deficiency (GHD). A possible involvement of these interactions in the pathogenesis of unexplained cases of GHD was suggested. Current research provides more and more details to the knowledge on the circadian rhythm of ghrelin. We gathered reports on the decreasing effect of *Helicobacter pylori*-related chronic gastritis on the number of ghrelin immunopositive cells and the consequent decrease in ghrelin serum concentration. The gastrointestinal tract microflora modification of the ghrelin action, by the mechanism of molecular mimicry, was also stressed. Moreover, the mutual relationships between ghrelin and the TSH-FT4/FT3 axis in growth and metabolic processes are described. It is to be recalled that FT4 and FT3 exert a permissive impact on IGF-1 action and, in turn, GH, in reaction mediated by IGF-1, enhances the monodeiodination of FT4 to FT3. Finally, we discussed the latest attempts to use the GH secretagogue receptor (GHS-R) analogues for possible diagnostic and therapeutic purposes.

## 1. Introduction

The problem of growth hormone (GH) deficiency (GHD), both in children and adults, is an interesting, and at the same time complicated and not fully understood phenomenon. For many years our study team have been conducting research on this subject, which resulted in many reports on human clinical studies and participation in international projects on GHD in adults [1,2,3,4,5,6,7,8,9,10]. Our further research is related to the elucidation of the not fully understood mechanisms leading to different severity and the expression of GHD in individual cases. Although it is known that deficiencies in the GH-insulin-like growth factor 1 (IGF-1) axis are a continuum from severe GHD to complete GH insensitivity [11], for which more than 90 different mutations of the GH receptor are described [12,13], certainly there are many non-genetically determined factors significantly affecting the regulatory mechanisms of the axis in question, and at least some of them seem to be modifiable.

More and more experimental and clinical data indicate that besides GH itself and IGF-1, also somatoliberin (GH releasing hormone (GHRH)), somatostatin (GH-inhibiting hormone (GHIH)), ghrelin, thyroid hormones and certainly many other factors are involved in the regulation of GH secretion and its growth-promoting effects (in children) and metabolic effects (in children and adults).

The benefits of using GH in the prevention of artheriosclerosis are increasingly emphasized. GH administration may be useful for improving endothelial dysfunction and it is generally known that this pathology enhances the development of artheriosclerosis. Further, GH ameliorates cardiac functioning after a myocardial infarction [14].

One of the causes of the isolated form of GHD is the so-called idiopathic GHD. The pathogenesis of this disease can be seen in the disturbances of the interactions among the different players mentioned above. These interactions were discussed in the present review.

## 2. Physiology of the GH/IGF-1 Axis

It is well-known that the production, secretion, and maintenance of the GH circadian rhythm depends on the stimulating effect of GHRH and the inhibitory effect of GHIH. Both these hormones are derived from the hypothalamus. Several neurotransmitters, neuropeptides, hormones and other factors are involved in the regulation of GH production and secretion, mainly indirectly via modulating GHRH and GHIH secretion. Among them, one of the most important hormones is ghrelin, which acts both indirectly, via the hypothalamus where it modulates the release of the mentioned hormones, and directly, via somatotrophs where it stimulates GH secretion [15,16]. The second important factor is IGF-1the main mediator of GH activity. Plasma IGF-1 can be used as a surrogate measurement of GH secretion. IGF-1 inhibits GH secretion, but the mechanism of this negative feedback is not clear [17].

Ghrelin is one of the strongest known GH secretagogues. It is produced and secreted mainly by X/A oxyntic mucosa endocrine cells of the stomach [18,19]. Two major forms of ghrelin were found in the circulation: unacylated ghrelin, which is the main circulating form, and acyl-ghrelin, which is the active form generated by otanoylation of the serine at position 3, a process mediated by ghrelin O-acyl transferase (GOAT) [20,21]. This acylation is essential for binding to the GHSR-1a and for most already known endocrine actions of ghrelin. Ghrelin secretion depends on age: in prepubertal children its concentration is higher than in pubertal ones and, generally, in children it is higher than in adults [22] (Table 1). However, there is no reference range for this hormone for each age group. Ghrelin secretion presents a circadian rhythm. In healthy humans, initial nocturnal elevation of ghrelin, declining towards the morning, is observed [23,24]. In turn, during the day, ghrelin secretion increases in the fasting state and decreases after meals. Ghrelin is also an orexigenic hormone, the secretion of which stimulates the feeling of hunger and consequent food intake [18,25].

Both total and acylated ghrelin levels can be determined in the serum. However, measuring acyl-grelin is a clinical challenge due to the fact that this molecule is easily degraded. One should be aware that it is necessary to properly prepare the blood sample for acyl-ghrelin collection (the blood sample must be collected in cold EDTA tubes, it is necessary to add a protease inhibitor and Hcl with minimal freezing and thawing) [26,27]. Therefore, many studies evaluate the total ghrelin concentration which reflects mainly the levels of desacylated ghrelin. However, Spiegel et al. [24] provided some evidence that acylated ghrelin is important mainly for appetite control and orexigenic effect but not for the GH-stimulating effect and that the acylated-to-total ghrelin ratio is lower during sleep than when awake, consistent with a reduction in orexigenic signal. Thus, in all our studies presented in this review, we assessed the total serum ghrelin levels.

Ghrelin is a natural ligand for GHS-R1a located on pituitary somatotrophic cells. Besides GHS-R1a, there is also isoform 1b of GHS R, which does not function as a receptor for ghrelin; however, it may attenuate activity of isoform 1a. GHS-R1a is a surface receptor belonging to the family of G protein-coupled receptors. 

**Figure 1:**
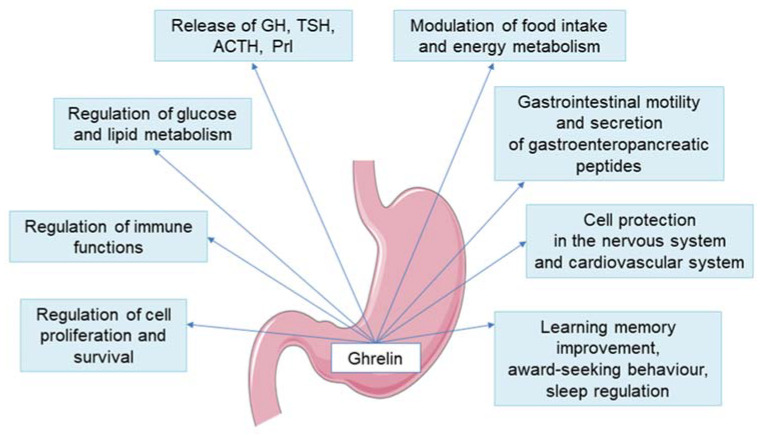
Directions of ghrelin effects on the functions of various systems and organs. ACTH—adrenocorticotropic hormone, GH—growth hormone, Prl—prolactin, TSH—thyroid stimulating hormone.

It is characterized by an intrinsic constitutive activity, namely the tonic signal generated that appears to be necessary for normal growth, possibly through its influence on the GH-IGF-1 axis [28,29]. Type 1a isoform binds mainly ghrelin, but it has also been demonstrated to bind both peptide [among others GH releasing peptide 6 (GHRP-6), macimorelin and non-peptide (e.g., ibutamoren) GH secretagogues [30]. By binding to the GHS-R1a receptor, ghrelin activates phospholipase C, leading to an increase in the concentration of inositol phosphate and the activation of kinase C. As a result, calcium ions are released from the endoplasmic reticulum. Thus, in the pituitary cells both non-endogenous and endogenous GHS-R1a agonists stimulate GH release in a manner dependent on intracellular Ca^2+^ concentration ((Ca^2+^) i). Similarly, in the arcuate nucleus GHS-R1a induces (Ca^2+^) i signaling in the neuropeptide Y (NPY)-neurons [31]. The receptor in question is widely distributed throughout the body and its expression was found in the nuclei of the hypothalamus, and also in the stomach, heart, lungs, kidneys, intestines, adipose tissue, as well as in many other locations. Figure 1 summarizes the directions of ghrelin effects on the functions of various systems and organs.

IGF-1 is the main peripheral mediator of GH activity and GHD is defined as the secondary IGF-1 deficiency [32]. It is produced mainly in hepatocytes; however, many other cell types are capable of synthesizing and secreting IGF-1. In addition to GH, a number of other factors influence the synthesis of IGF-1, such as nutrition and the immune system. Both IGF-1 bioavailability and the stability of its concentration are determined by binding to specific proteins, especially the insulin-like growth factor binding protein 3 (IGFBP-3) [17]. After binding to IGF-1, IGFBP-3 can bind acid labile subunit (ALS) to constitute a high-molecular-weight ternary complex. Similarly to GH, the highest rates of IGF-1 production occur during the pubertal growth spurt in adolescents and its synthesis lowers with age [31].

There are two receptors for IGF-1, namely IGF-1R and IGF-2R; both of them are surface receptors with intrinsic enzyme activity. The growth-promoting actions of IGF-1 is mediated through IGF-1R, which is structurally homologous to the insulin receptor. IGF-1R binds both IGF-1 and insulin; however, it binds insulin with 500–1000 times lower affinity. On the other hand, IGF-1 can also bind to and activate the insulin receptor. The IGF receptor differs functionally from the insulin receptor because it promotes primarily mitogenic/proliferative rather than metabolic activities. Under normal conditions, IGF-1 inhibit hypothalamic GHRH release and directly inhibits the synthesis of GH in pituitary somatotrophs in mechanism of negative loop feedback [17,33].

Ghrelin, GH and IGF-1 are three important players in the somatotropic axis; however, there are numerous differences among them, concerning the source of production, the model of secretion, the way of exerting their effects on both growing processes and metabolism, as well as their application in the diagnostics and treatment of GHD. Relationships between them are very complex.

## 3. Remarks on the Certain Limitations of GHD Diagnostics in the Transition Period from Childhood to Adult Age

GH deficiency means an impairment of GH secretion by the pituitary somatotroph cells. The condition may be hereditary or acquired, and GH deficiency may be isolated or combined with other pituitary hormone deficiencies (multiple hormonal deficiency, MPHD).

The most common cause of GHD in children is isolated GHD [32,34]. It should be stressed that only about 20% of children with GHD have an identifiable etiology. It includes mutations of the *GH-1* gene, the GHRH receptor gene [35] or genes encoding some transcription factors that take part in hypothalamo-pituitary region organogenesis (e.g., *HESX1*). In turn, mutations of other transcription factors (e.g., *PIT-1/POU1F1*, *PROP-1*, *LHX3*, *SOX2*, *SOX3*, *OTX2* and *HESX1*) lead to congenital MPHD [36], while hypothalamic-pituitary tumours (e.g., craniopharyngiomas), their treatment regimens or traumatic brain injuries result in acquired MPHD [34]. In many cases, a specific cause of GHD cannot be identified, and idiopathic GHD is diagnosed.

Regarding adults in whom GHD was diagnosed in childhood, the frequency of persistent severe GHD (CO-GHD) requiring further recombinant human GH (rhGH) treatment varies, ranging from as little as 10–20%, up to 70% of cases in isolated idiopathic GHD and nearly 100% of MPHD cases [9,37,38]. The above-mentioned differences in the frequency of persistent isolated GHD are alarmingly large and probably result from the differences in the applied diagnostic tests and their interpretation, which results in different criteria for the diagnosis of CO-GHD used by various authors. In the cohort of patients with isolated CO-GHD treated in childhood with rhGH preparations at the Department of Endocrinology and Metabolic Diseases, Polish Mother’s Memorial Hospital—Research Institute in Lodz, the frequency of persistent severe GHD after the end of the growth promoting therapy was assessed at 12.0% [9], while in the group of patients with morphologic lesions in the pituitary area (i.e., with pituitary stalk interruption syndrome [PSIS] or after neurosurgical procedures due to craniopharyngioma), persistent GHD affected all patients [1].

Thus, still almost 20% of adult patients [39,40] and over 70% of children with GHD constitute the cases of unknown etiology [34,36]. These data indicate that one should actively seek the causes of these unexplained cases of GHD in order to develop new diagnostic tools that will improve the diagnosis of GHD. It seems that among different backgrounds of GHD, attention should be given to possible disorders of ghrelin secretion; however, several other mechanisms of this phenomenon should also be considered.

It is to be recalled that the diagnosis of GHD in children and in adults is based on the clinical symptoms of GHD and reduced GH secretion in two (2) different stimulation tests after prior compensation of cortisol, thyroxine, and sex steroid deficiencies, if such deficiencies occur [39,40,41]. Regarding the diagnosis of GHD in adults it is sufficient to perform only one (1) stimulation test, and it is not necessary to perform tests in patients with a deficiency of at least three (3) other pituitary hormones and decreased serum IGF-1 concentration [42]. Both earlier recommendations from 1998 [37] and the last update from 2011 [41] suggest that two (2) different stimulation tests would be required to confirm the diagnosis of isolated GHD in adults. 

Also, during the transition of children with GHD to further rhGH treatment in adulthood (CO-GHD), the qualification is based on the results of two (2) stimulation tests for GH secretion, performed at least one month after the end of growth-promoting treatment [43]. The latest reports suggest the possibility of abandoning tests in patients with a deficiency of at least three (3) other hormones of the anterior pituitary gland and with morphologic lesions in the pituitary area (e.g., with PSIS or after neurosurgical procedures, due to, e.g., craniopharyngioma), as well as with confirmed mutations of the genes mentioned above [1,44,45,46]. In these cases, a decreased IGF-1 level at least one month after the end of rhGH therapy is considered to be a sufficient confirmation of severe GHD [44].

As mentioned before, only a small percentage of patients who undergo two (2) stimulation tests, are diagnosed with persistent GHD. The rest of the patients do not require rhGH treatment in adulthood because they are diagnosed with transient GHD. 

The cut-off points for normal and abnormal GH secretion in stimulation tests are arbitrary and vary between recommendations and countries [47,48,49,50,51]. These are obviously different for children and for adults because the amount of GH in the body needed for growth processes in a child is higher, and the amount of GH needed to exert metabolic effects in an adult is lower (Table 1). The principles of the GH stimulation tests and the different cut-offs proposed by different authors for diagnostics GHD in children and in adults are gathered in Table 2.

According to many authors, the currently used diagnostic methods are not fully satisfactory and should be replaced by new methods in order to make diagnosis more precise.

The need to expand knowledge in this area results from at least five important aspects:

(1) GHD in children is treated if the GHmax value in stimulation tests indicates not only severe GHD but also the so-called partial GHD (GHmax between 5 and 10 ng/mL) [49,50], while GHD in adults includes only cases with severe GHD (GHmax < 3 ng/mL according to the majority of authors). There are discussions on whether children with partial GHD should actually be treated with rhGH, as there are reports that they do not need such a therapy [52,53].
(2) Many authors express critical opinions concerning the usefulness, reproducibility and interpretation of the results of stimulation tests for GH secretion in children (a significant number of false-positive results) [54,55];
(3) There are limitations regarding the use of stimulation tests in adults (e.g., contraindications to the stimulation test after intravenous insulin administration in patients with ischemic heart disease, epilepsy, or in the elderly) [41,42] and differences in the obtained GH stimulation results, depending on the patient’s body mass index (BMI) [56];
(4) Most substances used as agents in the tests increase the secretion of GH indirectly, by stimulating the secretion of GHRH. There are only two (2) tests directly stimulating GH, namely the GHRH test and the test with use of macimorelin, which is a non-peptide synthetic GHS-R agonist [57]. Macimorelin received FDA approval for use in the diagnosis of GHD in adults in December 2018 [43,58] and it has not yet been registered for GHD diagnostics in children;
(5) The assessment of IGF-1 concentration is regarded as an important and even fundamental factor in the qualifying criteria for rhGH treatment [31]. However, IGF-1 deficiency can also be secondary to existing various chronic diseases, eating disorders and some inflammatory diseases [32].

In some cases, GHD may be not primary, but secondary to disturbances in the secretion of stimulating hormones, such as GHRH or ghrelin. In selected cases, the attempts to treat GHD with molecules stimulating the ghrelin receptor were performed [59,60,61]. Such treatment is tempting due to the ease of administration of the drug (orally instead of subcutaneously), however, the involvement of insufficient ghrelin stimulation in these cases is only one of the possible mechanisms. Certainly, many other factors may be involved in GHD pathogenesis in these cases.

## 4. New Insights into the Ghrelin and GH/IGF-1 Axis Integrating System

### 4.1. Disorders of Nocturnal Ghrelin Secretion

Maintaining the correct circadian rhythms of individual hormones is extremely important for human well-being, and their disturbances lead to significant clinical implications. Studies concerning the ghrelin circadian rhythm in adults and children are scarce. In 2011, Spiegel et al. [24] demonstrated their research concerning the ghrelin circadian rhythm carried out on healthy adult volunteers. The peak time (acrophase) of the circadian rhythm occurred 2 h after falling asleep in patients about 7 h 30 min after the dinner meal. After this peak, ghrelin levels declined until morning awakening at 07:00 a.m. and then increased before breakfast at 09:00 a.m. The morning ghrelin concentration was lower than that at the night-time acrophase. The positive correlations between nocturnal ghrelin and nocturnal GH secretion were confirmed in adults [62] and in children [63]. The question is whether night-time ghrelin secretion is reduced or altered in patients with GHD. Ghizzoni et al. [23] analysed circadian ghrelin concentrations in a sample of 15 prepubertal short children: 5 with GH neurosecretory dysfunction (NSD) and 10 with idiopathic short stature (ISS). The authors confirmed higher nocturnal than diurnal ghrelin concentration and showed that nocturnal GH secretion and IGF-1 concentration were lower, but nocturnal ghrelin was higher in GH NSD than in ISS children. A similar constellation, namely the lower the IGF-1, the higher the ghrelin concentration was observed in our analyses [63,64]. However, in our study which was published in 2020 [65], we decided to find out how the night-time ghrelin concentration measured in blood samples after 1 h and 1.5 h after falling asleep differed from the one in the early morning, the time routinely used for analysis in human research. In most studies, fasting ghrelin concentration is measured, and it is well-known that ghrelin levels increase in the morning, parallelly to the feeling of hunger. The reason for undertaking this research was the fact that the authors of most studies concerning GHD patients compare morning ghrelin concentration with peak GH secretion during GH stimulation tests and the results of these analyses are divergent [66,67]. We speculate that in patients with GHD or ISS there are significant discrepancies between nocturnal and morning ghrelin concentrations. However, based on our results, we confirmed that in individual patients both in GHD and ISS children, the morning ghrelin level changes parallelly to its nocturnal concentration; however, in the whole group of children the mean ghrelin concentrations were significantly higher than the nocturnal ones [65].

Thus, taking into consideration that the morning concentrations are affected by the feeling of hunger or satiety, it appears that nocturnal measurements better reflect the pool of ghrelin that is responsible for stimulation of GH and IGF-1 secretion. It remains to examine what factors reduce the nocturnal pool of ghrelin secretion and why, in some cases, ghrelin is unable to enforce the normal secretion of GH and IGF-1. It is known that nocturnal ghrelin increase is blunted during sleep deprivation and in obese patients [62]. It seems that the measurement of nocturnal ghrelin secretion can be helpful in determining the causes of GHD.

### 4.2. GHS Receptor Mutations Leading to Short Stature and GHD

Ghrelin exerts its effects through GHSR-1a. According to the data available from two large genome-wide association studies, a strong association between GHSR loci and height determination was confirmed [68,69]. As it was mentioned above, GHS-R1a is characterized by an intrinsic constitutive activity, the tonic signal generated in it appears to be necessary for normal growth, possibly through its influence on the GH-IGF-1 axis [29]. Interestingly, GHRH was also found to be an agonist of the GH-R1a [70].

Its blockage by inverse agonists or disturbation, resulting from genetic mutations or single nucleotide polymorphisms, is associated with family-related short stature or impairment of GH secretion with variable severity and penetrance [71,72,73]. Pantel et al. [71] described the missense mutation p.Ala204Glu in the second extracellular loop of the GHSR-1a in two unrelated patients with short stature. In the heterozygous state it was observed in a patient with isolated GHD, whereas in the homozygous state it was present in a patient with ISS. This mutation, which results in decreased cell-surface expression of the receptor, selectively impairs the constitutive activity of the GHSR while maintaining its ability to respond to ghrelin [71]. In turn, in 2009, Pantel et al. [74] reported an isolated GHD patient with delayed puberty who was heterozygote for two different GHSR mutations (p.Trp2X and p.Arg237Trp). GHD or ISS was also confirmed in patients with the following four novel heterozygous GHSR-1a mutations: p. Gln36del, p.Pro108Leu, p.Cys173Arg, and p.Asp246Ala, described by Inoue et al. [73]. Moreover, in 2011, Pugliese-Pires et al. [72], based on the analysis of patients with short stature and with or without constitutional delay of growth and puberty (CDGP), five different heterozygous point mutations in GHSR-1a were identified ((c.K6GOC, c.251GOT (p.Ser84Ile)), c.505GOA (p.Ala169Thr), c.545 TOC (p.Val182Ala), and c.1072GOA (p.Ala358Thr)), all in patients with CDGP [72]. Recently, Torz et al. [75] presented the results of their research on the significance of the GHSR-A203E mutation, and its correlation with the above-described GHSR-A204E mutation, which is associated in humans with short stature, GH deficiency and in some cases overweight and obesity. In short stature patients with A204E mutation, the loss of constitutive GHSR-1a activity was accompanied by retained ghrelin-dependent receptor activity. Mice expressing GHSR-A203E instead of the wild-type GHSR, showed also reduced body length and femur length and defective GH secretion in response to a 7 day, 60% caloric restriction. However, unlike humans, who were also overweight or obese, A203E mice had lower body weights than the wild-type animals. Importantly, GHSR-A203E mice showed a decreased appetite and ghrelin-induced GH release.

The authors concluded that the constitutive activity of GHSR contributed to native depolarizing conductivity in the arcuate NPY neurons. Moreover, they concluded that the GHSR-A203E mutation caused defective GH release, reduced length and body weight [75]. It is tempting to speculate that future genetic tests will reveal some disruptions in the transmission of ghrelin signals to the somatotropic cells and to GH secretion.

### 4.3. The Influence of Malnutrition on Ghrelin Secretion

It appears that ghrelin does not actually cause weight gain or loss, but rather is one of the components of the compensatory mechanism of energy homeostasis [74]. It was proved that in obese patients, ghrelin concentrations were reduced [75], while in patients with *anorexia nervosa* or with malnutrition, they were increased [76,77,78,79,80].

The diet-induced obesity suppressed the neuroendocrine ghrelin system by decreasing ghrelin production in the stomach, as well as by ghrelin resistance in arcuate NPY/AgRP neurons [81]. In those cases, ghrelin resistance was not limited to NPY/AgRP neurons, because ghrelin did not stimulate GH secretion in mice with diet-induced obesity [81]. 

It was observed in short stature non-GHD children that ghrelin concentrations differed depending on the body mass index. The concentrations were higher in slim children and lower in the obese ones. Simultaneously, it was observed that the values of IGF-1/IGFBP-3 molar ratio were significantly lower in slim patients than in the obese ones, while the GHmax concentrations, assessed by GH stimulation tests, were normal and comparable in both groups [82]. The mechanism of surviving starvation using a mouse model with an essential role of ghrelin and GH was described by Goldstein et al. [83]. Considering the fact that ghrelin secretion is increased in the condition of malnutrition, this mechanism can be understood as follows [84]: the increased ghrelin secretion in response to malnutrition causes an energy-saving effect through the influence of ghrelin on orexigenic centres. At the same time, the concentration of GH increases; it is also beneficial for the body, because, apart from augmentation of the growth processes and IGF-1 synthesis, GH is responsible for energy production by activating catabolism processes in the adipose tissue and the liver. When it comes to body fat, GH has a lipolytic effect; it triggers hydrolysis of triglycerides in adipose tissue, with the release of free fatty acids (FFA) into circulation. GH promotes cellular uptake of FFA by enhancing the activity of lipoprotein lipase in skeletal muscles [85,86]. Additionally, GH increases glucose production in the liver by enhancing glycogenolysis and gluconeogenesis. This phenomenon involves enhanced autophagy and an increased expression of the gluconeogenic genes, phosphoenolpyruvate carboxykinase and glucose-6-phosphatases [85,86]. Moreover, GH acting directly on carbohydrate metabolism exerts an effect antagonistic to insulin, whereas when acting indirectly via IGF-1 it reveals an insulin-like effect [85]. However, GH also exerts an anabolic effect; it regulates various physiological reactions, including muscle and bone anabolic processes [87,88]. The anabolic effects of GH are largely mediated by IGF-1 [85]. Thus, in this case, the effect of GH through IGF-1 would not be beneficial. 

Therefore, in a state of energy deficiency, GH is an important signal for mobilizing body fat and glycogen in order to maintain normal blood glucose levels. In turn, a hypothetical mechanism of GH resistance with respect to IGF-1 secretion may arise in order to maintain an elevated glucose concentration. 

Thus, in children with malnutrition (both qualitative and quantitative), a slow height velocity is observed, and this condition was reversed after recovery. An adverse effect of malnutrition on the growth process with blockage of IGF-1 secretion by sirtuin 1 (SIRT1) was described [89]. Sirtuins are a family of nicotinamide adenine dinucleotide (NAD+)-dependent enzymes [90]. Among seven of them (SIRT1–7), SIRT1 plays a key role in the organization and stabilization of the genome, in response to stress, glucose homeostasis, cell differentiation or oxidative damage [91]. It has also been found that SIRT1 can inhibit GH signalling by interacting with STAT3 and/or STAT5. By deacetylating of these molecules, it prevents their further phosphorylation, and consequently, their translocation to the nucleus [92,93].

GH acts through the GH receptor (GHR), which is a member of the class I cytokine receptor family. Although GHR is expressed ubiquitously, the liver is an organ most enriched in GHRs. The main pathway responsible for IGF-1 synthesis is Janus kinase 2, a signal transducer and activator of transcription (JAK2-STAT) pathway (Table 1). There are many disruptors of the GH signal transduction into a cell. Firstly, the mutations in the GH receptor or JAK2 and STAT5β genes result in a severe primary IGF-1 deficiency and short stature [94]. The malfunction of GHR-JAK2-STAT pathway may also result from the effects of many other modulators, some of them are not of genetic nature, but may depend on different eating habits or may result from the action of other modifiable factors. There are many post-receptor inhibitors of the GHR transduction pathway [89]. Among them, SIRT1 seems to be the most interesting protein.

The amount of SIRT1 depends on the availability and type of nutrients. In a fasting state or malnutrition, it intensifies the adaptive processes, i.e., gluconeogenesis and fatty acid oxidation, reducing the production of IGF-1, the factor which has a hypoglycaemic effect. Thus, SIRT1 negatively regulates GH-induced IGF-1 production (i.e., induces a GH-resistant state) [92]. Furthermore, an excess of sirtuins can lower IGF-1 levels, despite normal GH levels [95]. While no GH deficiency is found in these cases, the differential diagnosis of the observed IGF-1 deficiency should include malnutrition (qualitative and quantitative), as there may be cases in which GH stimulation tests are false positive.

However, the low IGF-1 and higher ghrelin are observed not only in malnourished children. In our previous studies concerning children with normal body mass and GHD or NSD, we observed that the lower the IGF-1 was, the higher the ghrelin concentration was. We proposed a tempting hypothesis that the low bioactivity of IGF-1 is a stimulating factor for ghrelin synthesis [63,64]. An interesting analysis was described by Oliviera-Santos et al. [96]. Authors calculated the area under the curve (AUC) of ghrelin in response to a mixed meal in a cohort of 20 adults with congenital, untreated, severe isolated GHD caused by homozygous mutation in the GHRHR gene (c.57+1 G→A) and in controls. In patients with GHD, higher AUC of ghrelin with less postprandial ghrelin attenuation and hunger attenuation, and with increased glucagon-like peptide 1 (GLP-1) secretion were observed. As ghrelin is a potent GLP-1 secretion enhancer, they speculated that high ghrelin secretion causes higher GLP-1 secretion, thereby contributing to the increased insulin sensitivity of subjects with IGHD. Additionally, the reduction in the somatostatin tone can contribute to the increase in GLP-1 in view of the feedback loop between GLP-1 and somatostatin [96,97]. Therefore, the authors’ results confirmed the existence of the mutual relationship between ghrelin, insulin/glucagon and GLP-1/GHIH. These correlations play an important role in the maintenance of carbohydrate homeostasis.

### 4.4. Limitation in the Production of Ghrelin in the Stomach: GOAT and Helicobacter Pylori

Considering that after bariatric surgery ghrelin level can be lowered by 80%, the effect of this condition on the functionality of the GH-IGF-1 axis should be carefully examined. 

**Figure 2:**
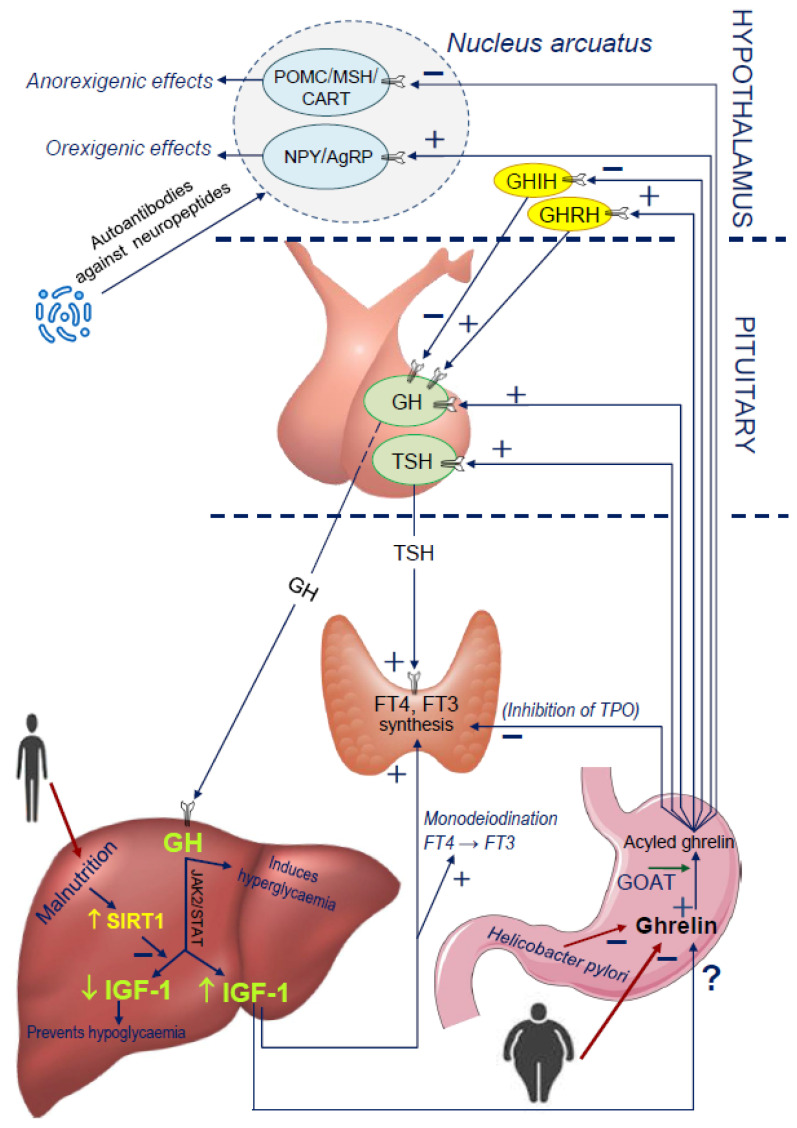
Reciprocal interactions among the main metabolic players (ghrelin, IGF-1) and TSH, thyroid hormone axis in the regulation of GH secretion. AgRP—Agouti-related peptide, CART—cocaine and amphetamine regulated transcript, FT3—free triiodothyronine, FT4—free thyroxine, GH—growth hormone, GHIH—growth hormone inhibiting hormone, GHRH—growth hormone releasing hormone, GOAT—ghrelin O-acyl transferase, IGF-1—insulin-like growth factor 1, MSH—melanocyte-stimulating hormone, NPY—neuropeptide Y, POMC—proopiomelanocortin, SIRT1—sirtuin 1, TPO—thyroid peroxidise, TSH—thyroid stimulating hormone.

As ghrelin is produced in the stomach, it is obvious that eating disorders, components of diet and microflora influence its production. After a meal rich in either carbohydrates or fat, the level of ghrelin decreases, while protein intake stimulates the secretion of ghrelin [98]. Firstly, the composition of the fatty acids, being the substrates for GOAT, in the daily diet, may influence the activity of this enzyme and the products resulting from its enzymatic action. This is a key process for fulfilling the biological function of ghrelin in CNS [20] (Figure 2).

Another limitation of the production of ghrelin in stomach cells is the *Helicobacter pylori* infection. Histologically, the human stomach consists of the cardia, body/fundus and antrum and is lined with gastric mucosa. Gastric mucosa of body and fundus is called oxyntic mucosa. In gastric mucosa there are gastric pits, surface invaginations that function as conduits of secretions; they are entirely lined with surface mucous (foveolar) cells regardless of the anatomic region. In the gastric mucosa there are also glands which are organized into isthmus, neck and base and they consist of different kinds of glandular cells. These cells synthesize acids, enzymes, mucins and hormones; the constituents and their products vary depending on the anatomic region of the stomach. Among them there are endocrine cells which are concentrated mainly within the mucous neck region. Their location varies depending on the type of products: G cells which produce gastrin, are limited to the antrum; while both D cells which produce somatostatin, and enterochromaffin (EC) cells which produce serotonin, are distributed all over the stomach. X/A-like cells are the second most abundant gastric endocrine cell type, accounting for 20–30% of oxyntic mucosa endocrine cells, ghrelin is a product of this type of cell [18] and they are mainly limited to the gastric body/fundus region [19]. In turn, *Helicobacter pylori* are located in gastric pits, mainly in the antrum.

The majority of analyses, concerning ghrelin concentrations in serum of *Helicobacter pylori* infected subjects vs. non-infected subjects, addressed the adult population. Gokcel et al. [99] demonstrated a lack of any correlation between ghrelin secretion rates in groups with or without *Helicobacter pylori* infection. However, their evaluation was, in that case, limited to adult female subjects with normal BMI [99]. In contrast, in other studies of adult subjects, significantly lower ghrelin concentrations were found in the blood serum of patients with *Helicobacter pylori* (+) vs. those with *Helicobacter pylori* (−) [100,101]. It has been reported in other publications that ghrelin concentration in *Helicobacter pylori*-affected subjects are reduced in the gastric juice only, remaining in serum within normal values [102,103]. It has also been demonstrated that significant effects on ghrelin secretion from the stomach in adult subjects are exerted by diseases, concomitant to *Helicobacter pylori* infection, such as peptic ulcer, gastric cancer and antral gastritis, or occurring without *Helicobacter pylori* infection, such as atrophic gastritis, affecting both the stomach fundus and the body [104].

Płonka et al. [105] conducted a study in children with normal height, and demonstrated that the levels of gastrin in the patients infected with *Helicobacter pylori* were significantly higher, whereas the levels of ghrelin and leptin were lower than in controls. In turn, Pacifico et al. 2008 [106] did not observe any significant differences in ghrelin concentrations in evaluated groups of children, either with or without *Helicobacter pylori* infection, although they observed a significant, inverse correlation between ghrelin concentration and histological severity of gastritis. These obvious inconsistencies may be a consequence of infection duration and the degree of concomitant inflammatory changes. It seems that different effects of *Helicobacter pylori* infection on ghrelin concentration are possible in children and adults, since stomach cells in children produce much more ghrelin than those in adult subjects. On the other hand, adult subjects demonstrate much more advanced inflammatory changes in gastric mucosa, which accompany *Helicobacter pylori* infection and peptic ulcers and/or atrophic inflammations of the whole stomach.

It is assumed that *Helicobacter pylori*-related chronic gastritis reduces the number of ghrelin immunopositive cells, suppresses ghrelin mRNA expression and decreases the ghrelin concentration in serum [107]. It has recently been reported that, after effective *Helicobacter pylori* eradication, ghrelin concentration rises, while leptin concentration falls and, additionally, appetite and body weight increase. 

The mechanism of molecular mimicry between *Helicobacter pylori* and a host that allows the manipulation of the host’s immune system is currently under investigation [108]. Perhaps, tests for *Helicobacter pylori* infection should be routinely performed during the diagnosis of GHD in children and adults. 

### 4.5. Potential Influence of Microbiota and Molecular Mimicry on Ghrelin Secretion

Recently, much attention has been focused on *microbiota*, microbes residing in the human body, which can influence our behaviour, mood and eating habits. A possible mechanism by which microbiota can exert these effects in the host is the modification of synthesis and activities of some enteropeptides due to the molecular mimicry phenomenon. Accordingly, a series of research reports concerning the phenomenon of molecular mimicry between the antigens of the intestinal microbiota and some regulatory peptides have been published [109,110,111]. The analysis of the amino acid sequence of these peptides showed the presence of homologous fragments with amino acid sequences of certain bacteria, viruses and fungi residing in the digestive tract. The phenomenon of molecular mimicry between *Helicobacter pylori* and leptin, insulin and αMSH, as well as between *Candida albicans* and ghrelin, leptin, insulin, NPY, orexin, αMSH and ACTH were suggested [109]. The authors showed that the presence of these microorganisms in the gastrointestinal tract can be a triggering factor for the production of antibodies that cross-react with neuropeptides and modify their regulatory action. We recently analysed the presence of selected neuropeptide (ghrelin, leptin, orexin A and αMSH) levels, as well as autoantibodies directed against them in 100 children (healthy and with short stature) with *Helicobacter pylori* infection and/or *Candida albicans* colonization [112]. We observed a significantly higher incidence of positive results (significantly higher concentrations of autoantibodies directed against selected neuropeptides) in children with short stature and *Helicobacter pylori* and/or *Candida albicans* than in children with normal height and with these infections (34.4% vs. 21.1%) [112]. In turn, the frequency of the presence of these antibodies in short stature children but without the gastrointestinal tract infections mentioned above, was reported at only 9.4%, while in the cases of children with normal growth and without these infections, such antibodies were not observed at all.

Our results revealed a relationship between short stature, the presence of some intestinal pathogenic microflora and the presence of autoantibodies directed against neuropeptides. Therefore, it is tempting to suspect a potential cross-reaction between antibodies against *Helicobacter pylori* and/or *Candida albicans*, and the neuropeptides involved in the growth process and food ingestion [112]. We also quantitatively analysed the concentration of four selected neuropeptides and antibodies directed against them in three (3) groups of children, with idiopathic short stature (ISS), with GHD and in the control group. Each group included children with *Helicobacter pylori* and/or *Candida albicans* infections, and children in whom the presence of these pathogens was not confirmed. We observed significantly higher levels of antibodies directed against ghrelin and leptin in children with ISS and *Helicobacter pylori* infection and/or *Candida albicans* than in the control group. On the basis of these results, one may conclude that the modification of the activity of ghrelin and leptin by autoantibodies directed against the hormones in question, can be responsible for impaired growth, poorer nutrition state and reduced IGF-1 concentration in these children [113,114], although this is only one out of many possible explanations (Figure 2).

### 4.6. Influence of TSH and FT4 on the Ghrelin-GH-IGF-1 Axis

It should be emphasized that relationships between ghrelin and many other hormones were also observed. It seems that mutual relations with thyroid hormones and TSH are especially interesting. Similar to GH and IGF-1, thyroid hormones regulate the linear growth of bones, protein synthesis, as well as the neuronal proliferation, migration and maturation. Similar to ghrelin, they also affect the basal metabolic rate (BMR).

Free T4 (FT4) and free T3 (FT3) exert a permissive impact on IGF-1 action; it was demonstrated that hypothyroidism, even in its subclinical form, affected IGF-1 secretion [115]. On the other hand, GH influences the monodeiodination of FT4 to FT3; this reaction is also mediated by IGF-1 [113]. In many children with GH deficiency, FT4 levels significantly decreased shortly after the beginning of GH replacement therapy [116,117,118,119,120]. It was proved that ghrelin is able to stimulate TSH secretion from thyrotropic cells of the anterior pituitary and the density of ghrelin receptors in mice seems to increase when the food intake is not sufficient [121] (Figure 2).

In most human studies in patients with hyperthyroidism and hypothyroidism, a positive correlation between ghrelin and TSH was confirmed [122,123]. However, this relationship does not only apply to persons with hyper or hypothyroidism and does not have to be related only to the patient’s metabolic state. Accordingly, a strong positive correlation between ghrelin and TSH was observed in euthyroid children with normal body mass and idiopathic (non-GH deficit) short stature [124]. Moreover, it was confirmed that the higher ghrelin and TSH were, the lower nocturnal GH secretion and lower the IGF-1 were. However, this observation was without any impact on FT4 concentrations [124]. Ghrelin receptors were found on human thyrocytes; the indirect suppression effect of ghrelin on thyrocytes was documented by Barington et al., in 2017 [125]. The cited authors concluded that ghrelin possesses the ability to reduce TSH-induced thyroglobulin level through the deterioration of thyroid peroxidase (TPO) expression [121]. Therefore, we may hypothesize that this is a reason why FT4 concentration is not elevated, despite higher TSH concentration.

We suppose that in children with ISS, in whom ghrelin concentration is elevated (probably in response to low IGF-1 secretion), TSH impact on FT4 secretion is reduced by higher ghrelin concentration. In the feedback mechanism, the increased secretion of TSH from the pituitary gland in order to normalize FT4 was observed [126].

The next hypothesis assumes that due to higher ghrelin concentration, the stimulation of TSH secretion from the pituitary gland is enhanced. However, due to a weaker impact of TSH on thyrocytes under this condition (high ghrelin concentrations), the FT4 level is not elevated and remains at the same level as in children with lower TSH.

It is possible that relative hypothyroidism disrupts the ghrelin-GHS-R axis impact on the stimulation of GH secretion, as GH secretion at night is disturbed in these children. At the same time, GH concentration obtained during GH stimulation tests, routinely used in GHD diagnostics, are normal [124].

## 5. Summary

In addition to the hypothalamic factors, such as the GHRH and GHIH, the GH-IGF-1 axis is influenced by ghrelin. Incorrect secretion and interaction of the above-mentioned hormones/factors may be conditioned by genetic background, but also by other modifiable factors, which should be taken into account in the diagnostics of a patient with suspected GH deficiency. These factors comprise eating disorders, gastrointestinal diseases, including *Helicobacter pylori* infection, abnormal composition of the intestinal microflora and improper diet, with both caloric and qualitative deficiencies (among others, affecting the operation of GOAT). In this jigsaw puzzle of various factors influencing the GH-IGF-1 axis, it seems that the proper concentration of thyroid hormones and TSH is also an essential element, extremely important for the adequate functioning of the axis in question, as well as for factors stimulating components of the above-mentioned axis, e.g., ghrelin.

Ghrelin is a pleiotropic hormone and disturbances in its action lead to many different abnormalities in the growth processes, as well as in food intake and metabolism. The knowledge about interactions of these hormones is important, especially that the methods of diagnosis of GHD proposed so far have not been entirely reliable (the reproducibility of stimulation tests for GH secretion is poor, and the concentration of IGF-1 is modified by numerous factors). Thus, new diagnostic methods that use ghrelin analogues (e.g., the macimorelin test) were recently proposed. Moreover, new drugs based on stimulation of the ghrelin receptor, especially non-peptide agonists, are at the stage of advanced clinical trials. 

Though the aim of considerations in the present review article was to indicate the significant role of ghrelin-related mechanisms in the pathogenesis of GHD in children; nevertheless, it seems that it is still far from the point where ghrelin agonists can become recognized drugs in the treatment of this disease.

However, taking into account all the facts and results of the research, it seems necessary to extend the diagnosis of idiopathic GHD and to include the following issues: (1) disorders of circadian rhythm of ghrelin secretion, (2) malnutrition, (3) *Helicobacter pylori* infections, (4) improper composition of dietary fats, (5) the presence of antibodies directed against elements of the intestinal microflora that interfere with the function of ghrelin in the CNS, (6) a positive correlation between the concentration of TSH and ghrelin and the possible modulating effect of ghrelin on the concentration of thyroid hormones.

Our present discussion was an attempt to analyse the cases of those patients who, at different times of their lives, were assessed differently as to possible growth hormone deficiency, in childhood they were qualified for this hormone treatment, and then in adulthood they did not fit into the group of patients, who, according to the accepted criteria, would still require this treatment.

The review includes two (2) main issues. The first is the imperfection of diagnostic tests and their incomplete reproducibility in a significant percentage of adults who were treated with GH before, in childhood. The second issue is the search for pathophysiological causes of this phenomenon. The possible participation of proper ghrelin functioning in the GH secretion mechanisms, as well as the mutual relationships between ghrelin and TSH-FT4/FT3 axis in growth and metabolic processes are described. It is generally known that FT4 and FT3 exert a permissive impact on IGF-1 action and in turn GH, in a reaction mediated by IGF-1, enhances the monodeiodination of FT4 to FT3.

On the basis of both our own results and the observations of other authors, we believe that the group of GHD patients includes individuals with ghrelin secretion disorders, possibly with interaction from the side of the hypothalamic-pituitary-thyroid axis.

The results of observations so far are not strong enough to make applications of ghrelin or its receptor analogues a recommended management in the treatment of GHD in some selected groups of patients. Nevertheless, we retain the position that in these people gastroenterological diagnostics should already be performed, comprising the secretion of ghrelin, as evidenced by the results of many clinical observations carried out over the last 20 years. It seems that the evolution of diagnostic tests for GH secretion (e.g., the macimorelin test) is also heading in this direction.

## Figures and Tables

**Table 1:** Characteristics of the structure of ghrelin, GH and IGF-1, their receptors and patterns of secretion.

	Ghrelin	GH	IGF-1
Chemical structure	Protein, 28 amino acids	Protein, 191 amino acids	Protein, 70 amino acids
Gene location	3p25.3	17q23.3	12q23.2
Main source of synthesis and secretion	Mainly the stomach X/A-like endocrine cells in oxyntic mucosa	Only somatotropic cells	Mainly hepatocytes
Circadian pattern of secretion	Maintained circadian rhythm: night—stable high concentration; day—increases in the fasting state and decreases after meal 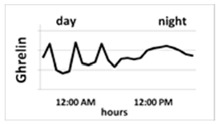	Maintained circadian rhythm: night—high peaks at III NREM phase; day—low concentration, with some peaks 3 to 5 h, lower than at night 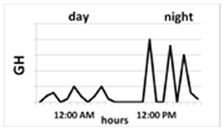	Without circadian rhythm, day and night are similar levels—stable, half-life is over 15 h 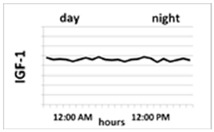
Lifetime secretion model	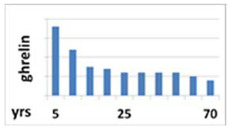	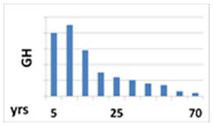	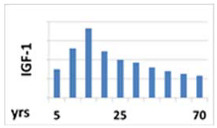
Name of receptor	GH secretagogues receptor (GHS-R)	GH receptor (GHR)	Insulin-like growth factor receptor (IGF-1R)
Gene location for the receptor	3q26.31	5p13-p12	15q26.3
Type of receptor	Surface, seven (7) transmembrane α helix hydrophobic domains, G-protein-coupled	Surface, class I cytokine receptor	Surface, receptor with intrinsic tyrosine kinase activity
Main pathway important for somatotropic axis	Intracellular calcium concentrations [Ca^2+^]_i_ signaling for GH secretion	JAK2-STAT for activation of IGF-1 gene	PI3K-AKT/PKB and the Ras-MAPK
Location of receptors	Pituitary somatotropic cells and the hypothalamus, also the stomach, heart, lungs, kidneys, intestines, adipose tissue and many other organs and tissues	GH receptors are most abundant in the liver	Ubiquitously

**Table 2:** The most commonly used tests stimulating GH secretion: methods of testing, dosing and cut-offs.

Stimulating Factor	Dosing	Time of Sample Collections (min)	Cut-Offs
Insulin	0.1 U/kg i.v.	−30, 0, 30, 60, 90, 120 (with simultaneous serum glucose tests)	3–5 ng/mL—Consensus, 1998 [37] 5.1 ng/mL—Biller, 2002; Molitch, 2011 [41,47] <3 ng/mL—Ho, 2007 [42] <5 ng/mL—Yuen, 2019 [43]
Glucagon	1 mg i.m. (in children 30 µg/kg body weight)	0, 90, 120, 150, 180 (glucose assessment every 30 min during the whole test)	<3 ng/mL—Ho, 2007, Yuen, 2019 [42,43] <1 ng/mL when BMI > 25kg/m^2^
L-DOPA	500 mg p.o.	−30, 0, 30, 60, 90	1.1 ng/mL; however, a test is not recommended due to the lack of adequate validation—Biller, 2002; Yuen, 2019 [43,47]
Arginine	0.5 g/kg i.v. for 30 min (maximum dose 30 g)	−30, 0, 30, 60, 90, 120	0.4 ng/mL; however, a test is not recommended due to the lack of adequate validation—Biller, 2002; Yuen, 2019 [43,47]
Arginine + GHRH	1 µg/kg i.v. bolus (GHRH), followed by a 30 min infusion of L-arginine (30 g)	−30, −15, 0, 30, 60	4.1 ng/mL, Biller, 2002 [47]
Clonidine	0.1–0.15 mg/m^2^, p.o.	−30, 0, 30, 60, 90, 120	<10 ng/mL—Wagner, 2014; Consensus, 2000; Murray, 2016 [48,49,50]
Clonidine + GHRH	0.15 mg/m^2^, p.o. (clonidine); 1 µg/kg i.v. bolus (GHRH), at time 60 min.	−30, 0, 30, 60, 75, 90,105, 120	<10 ng/mL—Devesa, 2017 [51]
Macimorelin	0.5 mg/kg, p.o.	−30, 0, 30, 60, 90, 120, 150	2.8 ng/mL—according to FDA, in adults, Yuen, 2019 [43]

## Abbreviations

ACTH	adrenocorticotropic hormone
ADH	vasopressin
AgRP	Agouti-related peptide
AMPK	adenosine monophosphate-activated protein kinase
AO-GHD	adult-onset growth hormone deficiency
BMI	body mass index
(Ca^2+^)I	intracellular Ca^2+^ concentration
CART	cocaine and amphetamine regulated transcript
CNS	central nervous system
CO-GHD	childhood-onset growth hormone deficiency
CRH	corticotropin-releasing hormone
ERK	extracellular signal-regulated kinase
ESE	European Society of Endocrinology
ESPE	European Society of Pediatric Endocrinology
FDA	Food and Drug Administration
FFA	free fatty acids
FT3	free triiodothyronine
FT4	free thyroxine
GLP-1	glucagon-like peptide 1
GH	growth hormone
GHD	growth hormone deficiency
GHIH	growth hormone inhibiting hormone
GHmax	maximal GH level during stimulation test
GHR	growth hormone receptor
GHRH	growth hormone releasing hormone
GHRP-6	growth hormone-releasing peptide 6
GHS-R	growth hormone secretagogue receptor
GHS-R1a	isoform 1a of growth hormone secretagogue receptor
GHS-R1b	isoform 1b of growth hormone secretagogue receptor
GOAT	ghrelin O-acyl transferase
GST	glucagon stimulation test
IGF-1	insulin-like growth factor 1
IGFBP-3	insulin-like growth factor binding protein 3
IRS	insulin-receptor substrates
ISS	idiopathic short stature
ITT	insulin tolerance test
IUGR	intrauterine growth retardation
JAK	Janus kinase
KIMS	Pharmacia and Upjohn International Metabolic Database
MAPK	mitogen-activated protein kinase
MCH	melanin-concentrating hormone
mRNA	messanger ribonucleic acid
αMSH	α-melanocyte-stimulating hormone
NAD	nicotinamide adenine dinucleotide
NPY	neuropeptide Y
NREM	non-rapid eye movement
PI3K-AKT/PKB	phosphatidylinositol 3-kinase and AKT/protein kinase B
PEPCK	phosphoenolpyruvate carboxykinase
POMC	proopiomelanocortin
Prl	prolactin
PSIS	pituitary stalk interruption syndrome
rhGH	recombinant human growth hormone
SIRT1	sirtuin 1
STAT	signal transducer and activator of transcription
TPO	thyroid peroxidase
TSH	thyroid stimulating hormone
